# Job satisfaction and determinant factors among midwives working at health facilities in Addis Ababa city, Ethiopia

**DOI:** 10.1371/journal.pone.0172397

**Published:** 2017-02-17

**Authors:** Eyasu Tamru Bekru, Amsale Cherie, Antehun Alemayehu Anjulo

**Affiliations:** 1 Department of Nursing, College of Health sciences and Medicine, Wolaita Sodo University, Wolaita Sodo, Ethiopia; 2 School of Public Health, Addis Ababa University, AddisAbaba, Ethiopia; 3 School of Medicine, College of Health sciences and Medicine, Wolaita Sodo University, Wolaita Sodo, Ethiopia; University of Ottawa, CANADA

## Abstract

**Background:**

Midwives are the primary source of care and support for mothers and newborns at the most vulnerable time in their lives.The Ethiopian National Reproductive Health Strategy targeted reduction of Maternal Mortality rate to 267/100,000 live births in the years 2006–2015. Midwives play a crucial role in the care of pregnant women, from the first antenatal visit right through to the delivery and the postpartum period.

**Methodology:**

Institution based cross-sectional study was carried out from March 2015 to April 2015 in Addis Ababa city, Ethiopia to assess job satisfaction and its determinants among midwives working at government health facilities. A total of 234 midwives were involved from 84 health centers and 8 governmental hospitals proportional to the size of health centers and hospitals using simple random sampling method. A total of 175 and 59 midwives were taken from health centers and government hospitals respectively. Different variables like Socio demographic, Job related domain and Organizational domain were collected using pre structured questionnaire after getting written consent. Data entry and analysis were done using SPSS 21.00. Binary logistic regression was used to determine factors affecting job satisfaction. P-values less than 0.05 were considered statistically significant.

**Result:**

From 234 eligible respondents 221 midwives participated in this study which makes a response rate of 94.44%. The overall mean job satisfaction was 52.9%. Independent predictors of job satisfaction includes Sex [AOR = 4.07 (95%CI: 1.36–12.37)], working unit [AOR = 0.04 (95%CI:(0.001–0.45)], Educational status [AOR = 5.74(95%CI: 1.48–40.47)], Marital status [AOR = 3.48 [1.01–11.97)], supervision [AOR = 4.33 (95%CI: 1.53–20.22)], standard of care[AOR 4.80, (3.38–50.10)] and work load [AOR 8.94, (95%CI 2.37–22.65)]. Midwives were least satisfied from salary, extrinsic reward and professional opportunity subscales while they were most satisfied from coworker relation and the standard of care they provided to clients.

**Conclusion:**

Half of study subjects were satisfied with their job. Governmental and Nongovernmental organizations should consider the factors that contribute to job dissatisfaction in order to improve service provision.

## Introduction

Health services organizations require highly qualified health professional to improve service provision of health care facilities in order to promote community health and to prevent diseases transmission [1, 2]. Midwives have been playing a pivotal role in the reduction of maternal and neonatal morbidity and mortality [3–5]. They are key actors in the achievement of Millennium Development Goal (MDG4) and MDG5 [6–7]. However, attainment of these goals in the least developed countries is still low(8). According to Ethiopian Demographic Health Survey (EDHS) 2011 report; Ethiopia Maternal Mortality Rate (MMR) continues in the high range of 676/100,000 live births [8].

Midwives are the primary source of care and support for mothers and newborns at the most vulnerable time in their lives. Almost every mother's birth experience and all forms of care in between are attended by midwives. Midwives can provide 87% of all basic sexual and reproductive as well as maternal and newborn health services [3]. They play vital role in the reduction of maternal and neonatal morbidity and mortality [4, 9]. Previous studies have shown that midwifery service could avert about two thirds of all maternal and newborn deaths. Achieving better maternal health requires quality reproductive health services and a series of well-timed interventions to ensure women’s safe passage to motherhood [3, 5].

Efficiency and productivity of human resources depends upon many factors, and job satisfaction is one of the most important factors [10]. The term job satisfaction refers to the attitude and feelings people have about their work [11].Job satisfaction of midwives has been a primary concern to health service organizations in both developed and developing countries. Job satisfaction is a pleasurable or positive emotional state from the appraisal of one's job or experiences. It is central to quality of maternal health care [9]. Low job satisfaction may result in increased turnover, tardiness, absenteeism, complaints and making the health care delivery system weak and extravagant. It may lead also to undesirable job performance and poor quality of service to clients [12–14].

Job Satisfaction can be affected by both external and internal factors. Job satisfaction among health professionals is derived from many interrelated factors and results in unintended output in health care service [15–16]. Every factor has its own importance and which cannot be neglected. It is known that reduction of maternal mortality was not achieved as planned in MDG. Level of motivation and level of satisfaction of job in health personnel could contribute for decreased achievement in stated MDG goal [8, 13–14]. More over Job satisfaction predicts job performance, staff morale, organizational citizenship, and quality of care, safety of patients and stability and effectiveness of an organization. Low job satisfaction leads to absenteeism, labor turnover and negative publicity of the organization. Unsatisfied workers are liabilities to any organization [13, 15]. It can affect the quality of service provision and number of clients who visits health facilities.

Job satisfaction of nurse measured by McClosky’s job satisfaction scale and satisfaction assumed if the score is greater than the mean of computed sub scale [3, 8, 14]. Other than demographic characteristics among nurses, satisfaction subscale and each containing different subscale to be rated by using likert score method are common variables in the study. The sub scales includes variables that may contribute for job satisfaction level of midwifes like extrinsic reward, prize and recognition, control and responsibility, coworker relation, interaction opportunity, scheduling and professional opportunity [12, 14, 17].

Shortage of midwives and high turnover resulting from low job satisfaction are the major impediments to achieve these goals [18–19]. Information from the Ethiopian Midwifery Data Base showed that Ethiopia has an estimated 4,725 midwives for a population of 85 million giving a ratio of 1: 17,989 which is inconsistent with WHO recommended midwife population ratio; which is 1:5000[9].

In recent years, a major target of the health care delivery system has been the provision of quality care to patients. A fundamental challenge, however in Ethiopia, still remains how to achieve improved patient outcome especially on maternal health care. As key members of the health care team, midwifes' job satisfaction plays an important role in the delivery of high-quality maternal health care along with other health professionals [20]. Factors like Level of motivation and satisfaction of job in health personnel were taken as challenge to achieve MDG goal [8, 13–14].

Factor affecting job satisfactions among health professional were varies from time to time and place to place [21]. Therefore, this study intended to assess level of job satisfaction and factors associated with level of job satisfaction among midwives working in hospitals and health centers under Addis Ababa city administration health bureau in Addis Ababa. Understanding level of job satisfaction and factors responsible for job satisfaction of midwives has supreme importance in the delivery of effective, efficient and sustainable services and reduction of maternal and neonatal morbidities and mortality [6, 8, 22–23].

## Methods

### Study area and period

Institution-based Cross-sectional study was carried out from March 10 to April 10, 2015 in Addis Ababa, Ethiopia. Addis Ababa is one of the regions and the federal capital of Ethiopia which lies at an altitude of 7,546 feet (2,300 meters). It covers a total area of 54,000 hectares. A total of 3,207,697 populations live in ten sub-cities distributed in 116 Weredas. Addis Ababa city administration health bureau consists of 116 health offices distributed in each Weredas and eight hospitals. Based on Ethiopia Service Provision Assessment and Census 2014; there are 84 health centers and a total of 6441 health professionals and technical staff distributed in each sub cities, contained in Arada sub city and Nifas silk sub city 8 health centers each, Addis ketema, Bole and KolfeKeranio sub cities 9 health centers each, Yeka sub city 11 health centers, Akake and kirkos sub city 7 health centers each, Lideta sub city 6 health centersandGulele sub city 10 health centers were providing health care for the public. Moreover there were eight government hospitals; Ras-Desta Hospital, Gandi Hospital, Minilk II Hospital, DagmawiMinilk Hospital, Zewditu Hospital, Yekatit Hospital, Hopco and Tirunshe Beijing Hospital with a total of 2686 supportive and professional staffs. The total population of midwives in hospitals and health centers under Addis Ababa city administration health bureau was 428

### Sample size

The sample size was calculated using a single population proportion to achieve a 95% confidence interval.We assumed 50% of the proportion of midwives job satisfaction, since no similar study was conducted in the past among midwives in Ethiopia with a 5% of margin of error and a 10% o non-response rate. However a total population of midwives in hospitals and health centers under Addis Ababa city administration health bureau was less than 10,000. Thus a total of 234 midwives were involved using correction formula from 84 health centers and 8 governmental hospitals using simple random sampling method; proportional to the size of health centers and hospitals. Based on proportional allocation a total of 175 midwives from health centers and 59 from government hospitals were distributed.

### Population

Study subject eligible for this study were Midwives working in government hospitals and health centers in Addis Ababa city for at least six month during the study period, who have academic rank diploma and above.

### Data collection instrument and procedure

Data were collected by highly qualified professionals using a semi structured self-administered questionnaires. Questionnaires were given to midwives working in health centers and government hospitals proportional to number of midwives present in the health facilities; 175 midwives from health centers and 59 from government hospitals participated in this study. The data collection instrument was adopted from a previous study [24]. Questionnaires were prepared and administered in English (S1 File). The questionnaire consisted of two parts. The first part which contains eight questions were used to assess the socio-demographic characteristics of respondents and the second part includes 55-item of job satisfaction subscale(31 McCloskey/Mueller Satisfaction Scale item)for measuring job satisfaction, and job and organizational dimension(25 items) of job satisfactions determinants. The assessment tools were scored by 5-point Likert scale (S1 File).

### Statistical analysis

Socio-demographic, job satisfaction and organizational dimension data entry and analysis were done using Epi data 3.1 and SPSS 21.00 version statistical software respectively. Each item of the overall job satisfaction were measured by a 5 point likert scale having a total of 31 items and their sum score ranging from a minimum of 31 to maximum of 155. Predictor items were also summated accordingly to determine agreement status of respondent by using computed mean for each sub scale and the higher means score indicating higher satisfaction from the subscale among the domains. Multivariable regression was used to adjust or control the possible confounding factors and to identify factors of job satisfaction. The cut point for Statistical significance was P < 0.05.

### Ethical statement

The study was approved by Ethics and Review Committee of Department of Nursing and Midwifery research ethics review board, College of Health sciences, Addis Ababa University. Letter of support was obtained from Addis Ababa city administration health bureau. Written consent was obtained from study participants in order to collect data.

## Results

### Socio demographic characteristics

Utilization of professionals’ data collectors with an academic degree of B.Sc maximized the response rate. From 234 eligible participants 221 provided the required data and giving the response rate 94.44%. From all study subjects 153(69.2%) were females and 68(30.8%) were males. Most of the respondents 148(66.9%) were single. Majority of the study subjects were younger age group, 185(207 (83.7%) were below 29 years of age with mean age 25.88 (±4.62) years. Of all 156 (70.6%) respondents had Diploma and 65(29.4%) bachelor’s degree (BSc)(Table 1).

**Table 1:** Background characteristics of midwives in government hospitals and health centers under Addis Ababa city administration health bureau, Addis Ababa Ethiopia, 2015 (N = 221).

Socio-demographic characteristics		Frequency	%
Age in years			
	< = 23	57	25.8
	24–28	128	57.9
	29–33	22	10.0
	>33	14	6.3
Sex			
	Male	68	30.8
	Female	153	69.2
Marital status			
	Single	140	63.3
	Married	72	32.6
	Divorced	9	4.1
Educational status			
	Diploma midwives	156	70.6
	BSc midwives	65	29.4
Working position			
	Staff midwives	193	87.3
	Head midwives	28	12.7
Work experience			
	< = 3	180	81.4
	4–6	28	12.7
	>6	13	5.9
Monthly Salary			
	< = 1994	138	62.4
	1995–2994	54	24.4
	>2995	29	13.1
Total		221	100

**Fig 1:**
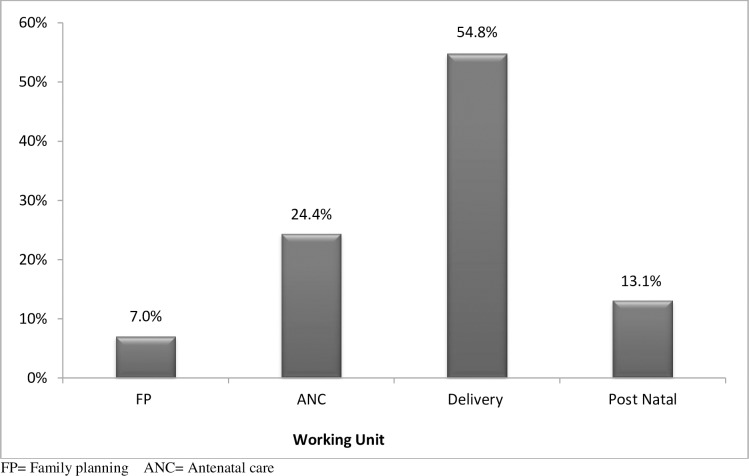
working unit distribution of Midwives, under Addis Ababa city administration health bureau, Addis Ababa Ethiopia, 2015.

Nearly half, 121(54.8%) of midwives were working in the delivery unit, 121(24%) in the ante natal clinic, 121(13.1%) in the post natal and 52(7.0) % in the family planning clinic(Fig 1).

#### Level of job satisfaction

Component of job satisfaction were assessed by 31 scales. Each item was classified as satisfied and unsatisfied by using component mean as cut of point. The items on the scale with which respondents were satisfied were significance of the job 179(81.0%), colleague relationship 164(74.2%), availability of maternity leave/other holiday leave 154(69.7%), opportunities to interact with other disciplines 153(69.2%), opportunities to participate in morning rounds 143(64.7%), recognition from head 139(62.9%), and opportunities for social contact 139(62.9%).

**Fig 2:**
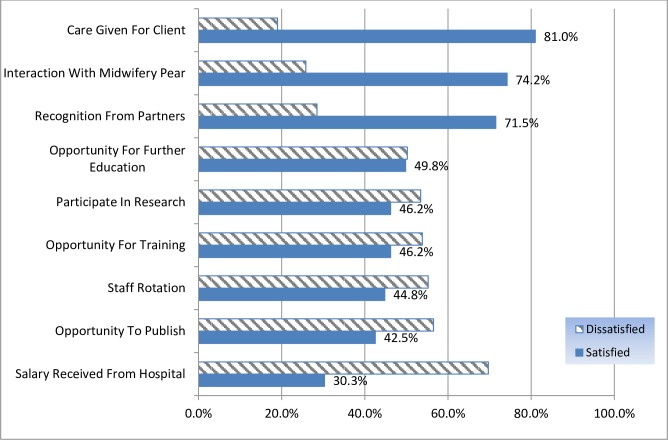
Dimension of job satisfaction items of top satisfying and dissatisfying scales among midwives in AA city administration government hospital and health centerEthiopia, 2015.

The items on the scale with which respondents were least satisfied were salary 154(69.7%). Staff rotation 122(55.2%), opportunities for part time work 121(54.8%), flexibility in scheduling 110(49.8%), compensation for working weekends & holidays, 119(53.8%) opportunities for on job training/short term training 119(53.8%) and opportunities for further education 111(50.2%) (Table 2) and (Fig 2).

**Table 2:** Frequency distribution of job satisfaction dimension items for satisfied and dissatisfied mxidwives under Addis Ababa city administration health bureau, Addis Ababa Ethiopia, 2015.

S.no	Component if job satisfaction sub scale	Satisfied		Dissatisfied	
		Frequency	%	Frequency	%
**Safety Domain**					
1.	Salary you receive from your hospital/ health center	67	30.3	154	69.7
2.	annual leave you receive from hospital/health center	121	54.8	100	45.2
3.	sick leave you receive from the hospital/health center	117	52.9	104	47.1
4.	hours that you work in the hospital/ health center	115	52.0	106	48.0
5.	flexibility in scheduling your working hours	122	55.2	99	44.8
6.	your satisfaction on staff rotation	99	44.8	122	55.2
7.	Opportunity for part-time work	100	45.2	121	54.8
8.	Flexibility in scheduling your weekends off	111	50.2	110	49.8
9.	Compensation for working weekends & Holidays	111	50.2	110	49.8
10.	Availability of maternity leave/other holiday leave	154	69.7	67	30.3
**Social Domain**					
1.	Recognition from your head for your work	139	62.9	82	37.1
2.	Interaction with your midwife peers/partners	164	74.2	57	25.8
3.	Interaction with the physicians you work with	155	70.1	66	29.9
4.	Availability of medical equipment’s/supplies	139	62.9	82	37.1
5.	Satisfaction with the midwifery care given to clients	179	81.0	42	19.0
6.	Opportunities for social contact	139	62.9	82	37.1
7.	Opportunities for interact with other disciplines	153	69.2	68	30.8
**Psychological Domain**					
1.	Opportunities for further education	110	49.8	111	50.2
2.	Opportunities to participate in morning rounds	143	64.7	78	35.3
3.	Opportunity to make autonomous care decisions	141	63.8	80	36.2
4.	Opportunities for on job training/short term training	102	46.2	119	53.8
5.	Recognition for your work from superiors	133	60.2	88	39.8
6.	Recognition for your work from peers/ partners	158	71.5	63	28.5
7.	Encouragement and positive feedback from your head	135	61.1	86	38.9
8.	Opportunities to participate in midwife research	102	46.2	119	53.8
9.	Opportunities to write and publish different publications	94	42.5	127	57.5
10.	Your responsibility in your unit/ward	169	76.5	52	23.5
11.	Your control over conditions in your working unit/ward	157	71.0	64	29.0
12.	Your amount of responsibility in hospital/health center	143	64.7	78	35.3
13.	Your participation in organization decision making	141	63.8	80	36.2
14.	Consideration given to your opinion and suggestions for change in the work	130	58.8	91	41.2

The mean scores of the eight subscales of job satisfaction were calculated and those responded below the mean of the subscale were considered as unsatisfied and those who are equal and above the mean were classified as satisfied. On the basis of these, four satisfaction and four dissatisfaction factors were identified. Among the satisfying factors Family/workplace balance 128(57,9%) min 2 max 10 mean 5.79±1.98, Coworker relation 131(59.3%) mean 6.84±, Interaction opportunities 117(52.9%) mean 4.24± 4.24 min 4 max 20 and control and responsibility 117(52.9%) Mean 19.67± 5.92 min 6 max 30.

Four of the dissatisfaction reported factors were professional opportunity 118(53.4%, extrinsic reward 117 (52.9%), scheduling 116(52.5%) and praise and recognition 113(51.1%)(Table 3).

**Table 3:** Job satisfaction Subscales Mean score among Midwives in government hospital and health center under Addis Ababa city administration health bureau, Addis Ababa Ethiopia, 2015 (N = 221).

Job satisfaction subscales	Min	Max	Mean	Std. D.	Satisfied		Dissatisfied	
					f	%	F	%
Extrinsic reward	3.00	15.00	7.87	3.06	104	47.1	117	52.9
Scheduling	5.00	25.00	13.44	4.32	105	47.5	116	52.5
Family/workplace balance	2.00	10.00	5.79	1.98	128	57.9	93	42.1
Coworker relation	2.00	10.00	6.84	2.38	131	59.3	90	40.7
Interaction opportunities	4.00	20.00	13.49	4.24	117	52.9	104	47.1
Professional opportunities	5.00	25.00	13.30	4.76	103	46.6	118	53.4
Praise and recognition	4.00	20.00	12.17	3.90	108	48.9	113	51.1
Control and responsibility	6.00	30.00	19.67	5.92	117	52.9	104	47.1

**Fig 3:**
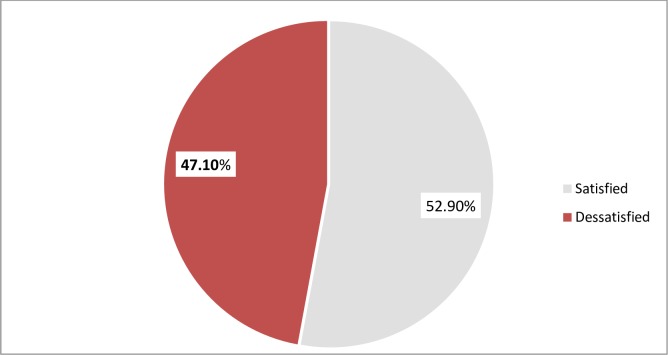
Overall of job satisfaction category among midwives in government hospital and health center under Addis Ababa city administration health bureau, Addis Ababa Ethiopia, 2015.

The overall job satisfaction was computed by using the mean score values of 31 job satisfaction facet scales. The highest potential score was 155, and the minimum potential score was 31 and he mean value of 92.64 ±22.53. On the basis of this only 52.9% of respondents reported satisfaction about their job(Fig 3).

**Fig 4:**
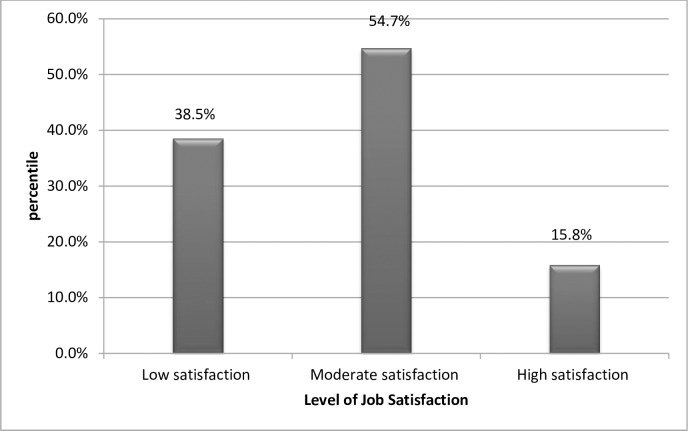
Level of job satisfaction among midwives in government hospital and health center under Addis Ababa city administration health bureau, Addis Ababa Ethiopia, 2015.

Levels of job satisfaction was determined by using data driven classification based on tertile classification rank order of overall job satisfaction score. The lower tertile rank corresponds to the low level of job satisfaction, the middle tertile corresponds to moderate and the upper tertile indicate the high level job satisfaction. Almost above one third (38.46%, n = 85) of the study participating midwives had low level of job satisfaction(Fig 4).

### Determinate of job satisfaction

Factors affecting job satisfaction which includes organizational and work domain were assessed by using 25 scales. Among respondents 175 (79.2%) agreed that the general standard of care given in the unit they work is good. In addition only 89 (40.3%) and78 (35.3%) of the respondents stated that they are satisfied by the work load and amount of responsibility respectively; which are all components of job related domain. From organizational domain items, among all respondents 172 (77.8%) agreed on receiving updates on equipment, forms, & protocols and only 103 (46.6%) agreed the fairness of payment for overtime and demanding hours. Nearly half of respondents (50.9%) have a pleasant attitude towards hospital/health center leadership(Table 4).

**Table 4:** Frequency distribution of job and organization related domain response of midwives in government hospital and health center under Addis Ababa city administration health bureau, Addis Ababa Ethiopia, 2015 (N = 221).

S.no	Job and organization related domain items	Agree		Disagree	
		F	%	F	%
1.	The Safety of environment you work in is good	111	50.2	110	49.8
2.	Do you get respect from management	105	47.5	116	52.5
3.	The communication among the team is good	153	69.2	68	30.8
4.	Promotions are Regular	155	70.1	66	29.9
5.	Consideration is given for experience in promotions	118	53.4	103	46.6
6.	Qualifications is considered for promotion	144	65.2	77	34.8
7.	Disruptions in social life due to working hours	122	55.2	99	44.8
8.	Work schedules is uncomfortable	130	58.8	91	41.2
9.	The standard of care given to the patient/client good	145	65.6	76	34.4
10.	The way that patient/ clients are cared for is good	135	61.1	86	38.9
11.	The supervisor observes you very closely	139	62.9	82	37.1
12.	The general standard of care given in this unit good	175	79.2	46	20.8
13.	patients are receiving the care that they need	173	78.3	48	21.7
14.	Have Sufficient time to get everything done	100	45.2	121	54.8
15.	Workloads in hospital/ health center proportional	89	40.3	132	59.7
16.	Payment for overtime and claiming hours is fair	103	46.6	118	53.4
17.	The type of leadership from your supervisor good	111	50.2	110	49.8
18.	You get to participate in supervisory decision that affects you	126	57.0	95	43.0
19.	payment goes along with over all activities in health center/hospital	127	57.5	94	42.5
20.	The supervisor is fair to you	134	60.6	87	39.4
21.	The supervisor observes you very closely	140	63.3	81	36.7
22.	Information regarding unit management is accessible	144	65.2	77	34.8
23.	Understand standard operating procedures and policy	155	70.1	66	29.9
24.	Receiving updates on equipment’s, forms, & protocols	172	77.8	49	22.2
25.	less amount of responsibility in the health center/hospital	78	35.3	143	64.7

Job and organization domain sub scales were also determined by using the subscale mean score after the scales under the sub scale were summated. Job related domain consist of five subscale which includes, working conditions, achievements, work itself, work load and standard of care and organizational domain consisted of three subscales including pay, Supervision and accessible administration policies(Table 5).

**Table 5:** Determinants of Job satisfaction Subscales Mean score among midwives in government hospital and health center under Addis Ababa city administration health bureau, Addis Ababa Ethiopia, 2015 (N = 221).

Subscales of job and organization related domain	Yes		No	
	F	%	F	%
1. Pleasant Nature of work	76	34.38	145	65.62
2. Fair Payment/salary	104	47.05	117	52.95
3. Conducive Working condition	106	47.96	115	52.04
4. Fair Supervision	107	48.42	114	51.58
5. Accessible administration &policies	111	50.22	110	49.78
6. Fair Workload	118	53.39	103	46.61
7. care to Standard	127	57.46	94	42.54
8. Better Achievements	135	61.09	86	38.91

Correlation analysis of job satisfaction, organization domains and job related domains were checked by Pearson Correlation. The relation between job satisfaction and standard of work was found to be significant with moderate strength. The other variables show weak correlation with job satisfaction(Table 6).

**Table 6:** Correlation analysis of job satisfaction, organization domains and job related domains among midwives in government hospital and health center under Addis Ababa city administration health bureau, Addis Ababa Ethiopia, 2015 (N = 221).

Variables	Job satisfaction	Workload	Working condition	Fairness of Supervision	Achievements	Administration policies	Fairness salary	Standard care
Workload	.333**	1						
Working condition	.286**	.194**	1					
Supervision	.269**	.141*	.083	1				
Achievements	-.146*	-.067	-.015	-.092	1			
Administration policies	.006	-.077	.170*	-.061	.035	1		
Salary	.308**	.073	.037	.057	-.191**	.014	1	
Standard care	.608**	.037	.209**	.220**	-.164*	-.022	.209**	1
Nature of work itself	-.064	-.085	-.130	.009	.028	-.119	.473**	.023

**. Correlation is significant at the 0.01 level (2-tailed).

*. Correlation is significant at the 0.05 level (2-tailed).

Bi-variable and multi variable logistic regression analysis was conducted to find the significant association of the dependent variable overall job satisfaction with the independent variable (sex, marital status, educational qualification, work experience and position/title of the midwives) and organizational and job related subscale. Correlation analysis preceded the logistic regression analysis to check for the existence of multi-collinearity. But none of the variables exceeded 0.70.

Multivariate logistic regression was done for variables that had statistically significant association with job satisfaction in crude analysis. The cut point for Statistical significance was P < 0.10 in Crude analysis. Females were about four times more likely to be satisfied than males [AOR = 4.70 (95%CI: 1.36–12.37)], educational status [AOR = 5.74(95%CI: 1.48–40.47)], and marital status [AOR = 3.48 [1.01–11.97)]. Significant association was also observed for working units. Those working in delivery unit were less likely to be satisfied than those working in other units[AOR = 0.04 (95%CI:(0.001–0.45)]. Those respondents who agreed by the fairness of supervision [AOR = 4.33 (95%CI: 1.53–20.22)] were around four times more likely to be satisfied in their job than the others. In addition perception of good standard of care [AOR 4.80, (3.38–50.10)] and fairness of work load [AOR 8.94,(95%CI 2.37–22.65)] were significantly associated with job satisfaction.

Other variables including, work experience, achievement, working condition, nature of work, accessible organizational policy and fairness of salary had shown no significant association with midwives job satisfaction(Table 7).

**Table 7:** Multivariable analysis for predictors of job satisfaction, Midwives, in government hospital and health center under A.A city administration health bureau, A.A. Ethiopia, 2015.

Independent Variables		Job Satisfaction		Crude OR [95% CI]	Adjusted OR [95% CI]
		Satisfied	Dissatisfied		
Age	24–28	73	55	1.47 [0.78–2.76]	0.74 [0.23–2.34]
	29–33	11	11	1.11 [0.41–2.97]	0.81 [0.10–6.69]
	>33	6	8	0.83 [0.25–2.71]	0.78 [0.5–11.61]
	< = 23	27	30	1	1
Sex	Female	94	59	3.11 [1.71–5.67]*	4.70 [1.34–16.48]**
	Male	23	45	1	1
Marital status	Married	39	33	0.99 [0.56–1.76]	3.48[1.01–11.97]**
	Divorced	2	7	0.24 [0.048–1.99]	1.56 [0.07–36.20]
	Single	76	64	1	1
Educational status	BSc-degree	47	18	3.2 [1.71–6.01]*	5.74[1.48–40.47]**
	Diploma	70	86	1	1
Works experience	3–6	12	16	0.60 [0.26–1.34]	2.13 [0.39–11.81]
	>6	5	8	0.50 [0.15–1.58]	0.16 [0.05–2.06]
	< = 3	100	80	1	1
Monthly salary	1995–2994	36	18	2.05 [1.06–3.97]*	0.34 [0.07–1.75]
	> = 2995	13	16	0.83 [0.37–1.87]	0.10 [0.01–1.24]
	< = 1994	68	70	1	1
Position	Staff	101	92	0.82 [0.37–1.83]	0.82 [0.15–4.36]
	Head	16	12	1	1
Working unite	ANC.	51	3	3.64 [0.66–20.06]	1.24 [0.09–16.48]
	Delivery	32	89	0.07 [0.02–0.28]*	0.04 [0.001–0.45]*
	Postnatal	20	9	0.47 [0.10–2.08]	0.36 [0.03–4.10]
	F.P	14	3	1	1
Nature of work itself	Pleasant	39	37	0.90 [0.51–1.57]	0.54[0.10–1.60]
	Unpleasant	78	67	1	1
Working condition	Conducive	59	47	1.23 [0.72–2.09]	0.37 [0.10–1.32]
	Not Conducive	58	57	1	1
Supervision	Fair	79	28	5.64 [3.15–10.8]*	4.33 [1.57–20.22]**
	Unfair	38	76	1	1
Achievement	Good	66	69	0.65 [0.38–1.13]	0.75[0.21–2.47]
	Poor	51	35	1	1
Workload	Low	89	29	8.22 [4.49–15.2]*	8.94 [2.37–22.65]**
	High	28	75	1	1
Fairness of salary	Fair	77	27	5.49 [3.06–9.81]*	4.26 [0.36–4.49]
	Unfair	40	77	1	1
Standard of care	Good	91	36	6.61 [3.64–11.9]*	4.80 [3.38–50.10]**
	Poor	26	68	1	1
Administration & policy	Accessible	58	53	0.94[0.55–1.60]	0.77 [0.22–2.76]
	Not accessible	59	51	1	1

Key

* = significant at P<0.005 in bivariate analysis.

** = significant at P<0.005 in multivariate analysis

FP = family planning, ANC = Anti Natal Care.

## Discussion

In most countries, midwifery is an additional qualification to the nursing qualification. So that many nurses are also midwives. Descriptions of the roles of nurses in Africa have usually been based on the opinions of expert groups such current roles of nurse/midwives in Sub-Saharan Africa health services, and the role expectations of stakeholders for nurses in Sub-Saharan Africa; the health, illness and care needs of the Sub-Saharan Africa population in the light of the type of health workers and their availability (skills mix) in the health team, with special reference to nurses.

In this study half of the participated midwives in this study were satisfied with their job. The overall job satisfaction in this study was 52.9%. This is almost comparable with previous studies in Ethiopia [19, 25] and a study in Islamabad [26]. Nonetheless, the job dissatisfactions by nearly half of the respondents are a reason for concern. Midwives play vital role in the provision of health services to mothers and newborns at the most critical period in their lives. In addition, institutional mother and new born care is one of the strategies to reduce maternal and neonatal mortality and to achieve MDG. Thus, Job dissatisfaction among midwives has effect on the efficiency and effectiveness of the service and may retract the achievement of reduction of maternal and neonatal mortality.

This study pinpointed that more than half of midwives in this study were dissatisfied by extrinsic reward, scheduling, absence of praise and recognition and professional opportunity. Provision of opportunities for further education and training both short term and long term is imperative for amplified motivation and maximized satisfaction and improving the knowledge and skill of midwives. In addition, recognition of the work done, acknowledgments of outstanding efforts and good performances and proper balance and fairness in scheduling can heighten the morale of midwives and intensify the quality of service [27–29].

High job satisfaction was observed from good interpersonal relationships with co-workers which is similar to Lebanon and Malaysian studies [28,30]. This should be encouraged since good interpersonal relationship and teamwork has impact in the service rendered. From job and organization domains the list satisfied sub scale in this study was pay which was in line with a study done in Jimma specialized hospital south west Ethiopia among health professionals, nurses in sidama zone south Ethiopia and descriptive study design to assess job satisfaction of registered nurses in South Africa[13,19,31]. But a survey of registered nurse in USA, and descriptive study carried out on job satisfaction among nurses in India, showed slight satisfaction for payment [32–33]. This might be due to insufficient salary compared to the task and responsibility given for midwives.

From multi variable analysis it was observed that Female respondents were about three times more likely to be satisfied than male respondents. This was congruent with a study conducted in USA [34] and in line with a cross sectional study in Jimma which showed more dissatisfaction among males [13] but different from a study made on male nurse working in Nigeria where high satisfaction was observed among males. This discrepancy might be from perception about the nature of the work especially in working in labor ward and the societal believes that midwifery is mostly female’s profession. There are also literatures describing old English literal interpretation of midwifes as “with women” which may have negative feeling among male professionals. It is also part of most history that Traditional birth attendant are female mother. Married midwifes were more satisfied than those who are not. The same result was observed among those nurses in Sidam zone. These might be due to stability in life and emotional situation and encouragement from marriage partners. The same was also observed with Nigerian nurses [35].

Midwives with BSc were more likely to be satisfied by their job than those who are diploma holders. This contradicts with a study in USA and Kuwait which showed nurses with higher level of education were dissatisfied by their job. But a study in England found that nurses without updates and education are less knowledgeable and less motivated [36]. The difference might be due to limited numbers of midwives are available in Ethiopia which makes increased opportunity for part time work and create good carrier opportunity.

Midwives working in delivery unit were 40% less likely to be satisfied than those working in other units. This might be due to the invasiveness of the procedure in delivery room which increases the risk of contamination, stress and tiredness. More over providing care for laboring mother requires conscious, alert state of mind and extended hour of care which might predispose to excessive tiredness. This is also supported by a study done in Senegal which indicated that midwives were dissatisfied with their working conditions, remuneration, and extremely high levels of tiredness [10].

Those who agreed with fairness of supervision were about four times more likely to be satisfied than their counterparts. This result is inconsistent with a study done in Pakistan among public health workers which showed dissatisfaction with supervision [37]. The result in this study might be due to supportive supervision which makes workers and supervisors to work together. Supportive supervision makes workers to like their working environment and, improve their efficiency, which in turn will increase job satisfaction. This is similar with a study done in Canada [38].

In this study respondent midwives who reported fair work load were 8 times more likely to be satisfied than those who were overloaded. Increased work load limits achievement, communication time with patients and quality of care to be provided.

Participants who perceived high level of patient care were 6 times more likely to be satisfied with their job than those who reported poor standard of care. This is different from Pakistanis public health worker who showed no significant association between job satisfaction and standard of service they were providing to the client [38]. The possible explanation could be helping a person in pain may give satisfaction which was evidenced by a study in Jimma specialized hospital health workers which showed that helping the patient was the main reason for their job satisfaction.

## Conclusion and recommendation

This study revealed that only half of midwives were satisfied with their job. More over the satisfaction have shown significant association with those factors including sex, education status, marital status, working unit, coworker interaction, supervision, standard of care and work load. More than half of respondents were dissatisfied by extrinsic reward, scheduling, absence of praise and recognition, professional opportunity and salary.

## Supporting information

S1 File Annex I: Consent form. Annex II: Questionnaire. Annex III: Descriptive Result Table. Annex IV: Questionnaire description.
(DOCX)
