# Supplementary material for: Job satisfaction and determinant factors among midwives working at health facilities in Addis Ababa city, Ethiopia
Source: PLoS One. 2017 Feb 17;12(2):e0172397. doi: 10.1371/journal.pone.0172397 (PMC5315386; doi:10.1371/journal.pone.0172397)
Supplement: S1 File — Annex I: Consent form. Annex II: Questionnaire. Annex III: Descriptive Result Table. Annex IV: Questionnaire description. (DOCX) [file pone.0172397.s001.docx]

**Supporting information**

**Appendix** I: Consent form

ADDIS ABABA UNIVERSITY COLLEGE OF ALLIED HEALTH DEPARTMENT OF NURSING AND MIDWIFERY

A QUESTIONNAIRE TO ASSESS FACTORS ASSOCIATED WITH LEVEL OF JOB SATISFACTION AMONG MIDWIVES IN GOVERNMENT HOSPITALS AND HEALTH CENTERS UNDER ADDIS ABABACITY ADMINISTRATION HEALTH BUREAU, ADDIS ABABA 2015.

**Consent Form for the Study**

I have read the information on the title and aim of the study given above. The title and aim of the study was clear to me. I understood that participation in this study is completely voluntary and that if I want to withdraw from the study any time, I will not obliged to continue and I will withdraw from it at any time. My answers are confidential and my name is not required on any documents. I understand no one other than the investigator and his advisors will have access to the questionnaire at any time. I understand that there is no risk associated with participating in this study and also no direct benefits, but the result may help him in obtaining the best information possible from nurses working in Addis Ababa public hospitals and health centers.

So, I agree to this, provided that my privacy is guaranteed .I hereby give informed written consent to participate to this study.

Name of the hospital/health center _________________

Signature of volunteer _______________ Date ______________

**Appendix II: Questionnaire**

**Instructions:**

1. **You are kindly requested to answer the following questions.**
2. **Encircle the answer of your choice regarding each question.**
3. **For certain questions when additional information is needed, please write your answer on the space provided.**
4. **Please do not write your name on any page of the paper.**

**Part I: General information (socio demographic characteristic of the participants).**

| No. | Socio demographic and Employee profile questions | Coding categories |
| --- | --- | --- |
| Socio-demographic factors | | |
| **101** | Sex | 1. Male 2. Female |
| **102** | Your Age in years | ____ years |
| **103** | What is your current marital status? | 1. Married 2. Single 3. Divorced 4. Widowed |
| **104** | Length of service /Your working experience in the hospital/health center (in years) | _____Years |
| **105** | Your educational qualification | 1. Diploma midwife 2. BSc midwife 3. MSc midwife 4. Other(Specify__________ ) |
| **106** | Your current salary | ____________________ |
| **107** | Which hospital/health center are you working? | 1. _________________________ |
| **108** | \| Current Working  Unit/ward \| \| --- \| | 1. Family planning 2. ANC room 3. Delivery Room 4. post natal ward 5. Neonatal care unite 6. Gynecological ward 7. Gyn/Obs OPD 8. other _______________ |
| **109** | What is your title? Or Position that you presently hold within the hospital | 1. Staff midwife 2. Head midwife 3. Supervisor midwife 4. Other _____________ |

**Part II: job Satisfaction questionnaire based on MMSS and job/organization factor.**

The following questions measures your job satisfaction levels and factors that contribute for being satisfied or being dissatisfied as a midwife personal in the hospitals/health center you working in.

How satisfied are you with the following aspects of your current job?

Please **circle** the number that applies your current satisfaction level in front of each question/item.

**Dimensions of job satisfaction**

| 1 = Very  Dissatisfied | | 2 =Moderately  Dissatisfied | 3= neutral | 4 = Moderately  Satisfied | | | 5= Very  Satisfied | | | |
| --- | --- | --- | --- | --- | --- | --- | --- | --- | --- | --- |
| 201 | Pay (Salary) you receive from your hospital as a midwife | | | | 1 | 2 | | 3 | 4 | 5 |
| 202 | Annual leave you receive from the hospital/health center | | | | 1 | 2 | | 3 | 4 | 5 |
| 203 | Sick leave you receive from the hospital/health center | | | | 1 | 2 | | 3 | 4 | 5 |
| 204 | Hours that you work in the hospital/ health center | | | | 1 | 2 | | 3 | 4 | 5 |
| 205 | Flexibility in scheduling your working hours | | | | 1 | 2 | | 3 | 4 | 5 |
| 206 | Your satisfaction in your shift rotation | | | | 1 | 2 | | 3 | 4 | 5 |
| 207 | Opportunity for part-time work | | | | 1 | 2 | | 3 | 4 | 5 |
| 208 | Flexibility in scheduling your weekends off | | | | 1 | 2 | | 3 | 4 | 5 |
| 209 | Compensation for working weekends & Holidays | | | | 1 | 2 | | 3 | 4 | 5 |
| 210 | Availability of maternity/holyday and related leave | | | | 1 | 2 | | 3 | 4 | 5 |
| 211 | Recognition from your head for your work | | | | 1 | 2 | | 3 | 4 | 5 |
| 212 | Interaction with your midwife peers/partners | | | | 1 | 2 | | 3 | 4 | 5 |
| 213 | Interaction with the physicians you work with | | | | 1 | 2 | | 3 | 4 | 5 |
| 214 | Availability of medical equipment/supplies to deliver quality midwifery care in your unit | | | | 1 | 2 | | 3 | 4 | 5 |
| 215 | Satisfaction with the nursing care given to your clients | | | | 1 | 2 | | 3 | 4 | 5 |
| 216 | Opportunities for social contact with your colleagues after work | | | | 1 | 2 | | 3 | 4 | 5 |
| 217 | Opportunities for interact professionally with other disciplines | | | | 1 | 2 | | 3 | 4 | 5 |
| 218 | Opportunities for further education/degree or post graduate in midwifery | | | | 1 | 2 | | 3 | 4 | 5 |
| 219 | Opportunities to participate in morning rounds | | | | 1 | 2 | | 3 | 4 | 5 |
| 1 = Very  Dissatisfied | | 2 =Moderately  Dissatisfied | 3= neutral | 4 = Moderately  Satisfied | | | 5= Very  Satisfied | | | |
| 220 | Opportunity to make autonomous midwifery care decisions | | | | 1 | 2 | | 3 | 4 | 5 |
| 221 | Opportunities for on job training/short term training | | | | 1 | 2 | | 3 | 4 | 5 |
| 222 | Recognition for your work from superiors | | | | 1 | 2 | | 3 | 4 | 5 |
| 223 | Recognition for your work from peers/ partners | | | | 1 | 2 | | 3 | 4 | 5 |
| 224 | Encouragement and positive feedback received from your matron | | | | 1 | 2 | | 3 | 4 | 5 |
| 225 | Opportunities to participate in midwife research | | | | 1 | 2 | | 3 | 4 | 5 |
| 226 | Opportunities to write and publish | | | | 1 | 2 | | 3 | 4 | 5 |
| 227 | Your responsibility in your unit/ward | | | | 1 | 2 | | 3 | 4 | 5 |
| 228 | Your control over conditions in your working unit/ward | | | | 1 | 2 | | 3 | 4 | 5 |
| 229 | Your amount of responsibility | | | | 1 | 2 | | 3 | 4 | 5 |
| 230 | Your participation in organization decision making | | | | 1 | 2 | | 3 | 4 | 5 |
| 231 | Consideration given to your opinion and suggestions for change in the work setting or office practice | | | | 1 | 2 | | 3 | 4 | 5 |

**Instruction: - please read each item and give your fair response by the rank given in the box.**

**Job and organizational domain**

| 1 = very disagree | | 2 = disagree | 3= neutral | 4 = Agree | | | 5= very agree | | | |
| --- | --- | --- | --- | --- | --- | --- | --- | --- | --- | --- |
| 232 | The Safety of environment you work in is good | | | | 1 | 2 | | 3 | 4 | 5 |
| 233 | Do you get respect from management | | | | 1 | 2 | | 3 | 4 | 5 |
| 234 | The communication among the team is good | | | | 1 | 2 | | 3 | 4 | 5 |
| 235 | The type of leadership from your supervisor good | | | | 1 | 2 | | 3 | 4 | 5 |
| 236 | The supervisor observes you very closely | | | | 1 | 2 | | 3 | 4 | 5 |
| 237 | The supervisor is fair to you | | | | 1 | 2 | | 3 | 4 | 5 |
| 238 | You get to participate in supervisory decision that affects you | | | | 1 | 2 | | 3 | 4 | 5 |
| 239 | Promotions are Regular | | | | 1 | 2 | | 3 | 4 | 5 |
| 240 | Consideration is given for experience in promotions | | | | 1 | 2 | | 3 | 4 | 5 |
| 241 | Qualifications is considered for promotion | | | | 1 | 2 | | 3 | 4 | 5 |
| 242 | Workloads in hospital/ health center is proportional | | | | 1 | 2 | | 3 | 4 | 5 |
| 243 | Disruptions in social life due to working hours | | | | 1 | 2 | | 3 | 4 | 5 |
| 244 | Work schedules is uncomfortable | | | | 1 | 2 | | 3 | 4 | 5 |
| 245 | Payment for overtime and claiming hours is fair | | | | 1 | 2 | | 3 | 4 | 5 |
| 246 | Do you have Sufficient time to get everything done | | | | 1 | 2 | | 3 | 4 | 5 |
| 247 | Understand standard operating procedures and policy | | | | 1 | 2 | | 3 | 4 | 5 |
| 248 | Receiving updates on equipment’s, forms, & protocols | | | | 1 | 2 | | 3 | 4 | 5 |
| 249 | Information regarding unit management is accessible | | | | 1 | 2 | | 3 | 4 | 5 |
| 250 | payment goes along with over all activities in health center/hospital | | | | 1 | 2 | | 3 | 4 | 5 |
| 251 | The way that patient/ clients are cared for is good | | | | 1 | 2 | | 3 | 4 | 5 |
| 252 | The standard of care given to the patient/client good | | | | 1 | 2 | | 3 | 4 | 5 |
| 253 | You are currently able to give standard of care | | | | 1 | 2 | | 3 | 4 | 5 |
| 254 | The general standard of care given in this unit good | | | | 1 | 2 | | 3 | 4 | 5 |
| 255 | Patients are receiving the care that they need | | | | 1 | 2 | | 3 | 4 | 5 |

**Thank You for your participation!**

**Appendix** III**:** Descriptive Result Table

Ranking order by mean for job satisfaction scales of midwives in government hospital and health center under Addis Ababa city administration health bureau, Addis Ababa Ethiopia, 2015.(n=221)

| rank | Dimensions of job satisfaction sub scale | Min | Max | Mean | Std. D. |
| --- | --- | --- | --- | --- | --- |
|  | (Salary) you receive from your hospital/ health center as a midwife | 1 | 5 | 2.23 | 1.336 |
|  | Opportunities to write and publish different publications | 1 | 5 | 2.47 | 1.219 |
|  | Opportunities for on job training/short term training | 1 | 5 | 2.49 | 1.306 |
|  | Flexibility in scheduling your weekends off | 1 | 5 | 2.53 | 1.216 |
|  | Opportunities to participate in midwife research | 1 | 5 | 2.56 | 1.389 |
|  | Opportunity for part-time work | 1 | 5 | 2.58 | 1.355 |
|  | Compensation for working weekends & Holidays | 1 | 5 | 2.61 | 1.207 |
|  | Opportunities for further education/degree or post graduate in midwifery | 1 | 5 | 2.65 | 1.523 |
|  | Your satisfaction in your shift rotation | 1 | 5 | 2.76 | 1.329 |
|  | that you work in the hospital/ health center | 1 | 5 | 2.77 | 1.380 |
|  | Flexibility in scheduling your working hours | 1 | 5 | 2.78 | 1.250 |
|  | leave you receive from the hospital/ health center | 1 | 5 | 2.80 | 1.299 |
|  | leave you receive from the hospital/ health center | 1 | 5 | 2.85 | 1.284 |
|  | Recognition for your work from superiors | 1 | 5 | 2.87 | 1.255 |
|  | Consideration given to your opinion and suggestions for change in the work | 1 | 5 | 3.00 | 1.378 |
|  | Recognition from your head for your work | 1 | 5 | 3.02 | 1.352 |
|  | Encouragement and positive feedback received from your matron | 1 | 5 | 3.04 | 1.460 |
|  | Your participation in organization decision making | 1 | 5 | 3.10 | 1.373 |
|  | Opportunity to make autonomous midwifery care decisions | 1 | 5 | 3.12 | 1.298 |
|  | Opportunities to participate in morning rounds | 1 | 5 | 3.13 | 1.425 |
|  | Opportunities for social contact with your colleagues after work | 1 | 5 | 3.16 | 1.436 |
|  | Availability of medical equipment’s/supplies to deliver quality care in yo | 1 | 5 | 3.19 | 1.401 |
|  | Availability of holiday leave /maternity leave | 1 | 5 | 3.21 | 1.370 |
|  | Recognition for your work from peers/ partners | 1 | 5 | 3.24 | 1.230 |
|  | Opportunities for interact professionally with other disciplines | 1 | 5 | 3.28 | 1.386 |
|  | Interaction with the physicians you work with | 1 | 5 | 3.33 | 1.329 |
|  | Your control over conditions in your working unit/ward | 1 | 5 | 3.34 | 1.271 |
|  | Your amount of responsibility | 1 | 5 | 3.52 | 1.387 |
|  | Interaction with your midwife peers/partners | 1 | 5 | 3.55 | 1.360 |
|  | Your responsibility in your unit/ward | 1 | 5 | 3.57 | 1.269 |
|  | Satisfaction with the midwifery care given to your clients | 1 | 5 | 3.87 | 1.320 |

# Appendix IV: Questionnaire description

| Parts | Item coding category | Number of questions | Subscale category |
| --- | --- | --- | --- |
| Part-I | Item 101 – 108 | 8 | -Socio demographic characteristic |
| Part-II | Part-II has eight sub scales with 31 items which measures job satisfaction based on MMSS. |  | - |
|  | Item: 201, 202, 203 | 3 | Extrinsic reward |
|  | Item: 204,205,206,208 & 209 | 5 | Scheduling |
|  | Item: 207,210 | 2 | Family/workplace balance |
|  | Item: 212, 213 | 2 | Coworker relation |
|  | Item: 214,215,216 & 217 | 4 | Interaction opportunities |
|  | Item: 218,219,221,225 & 226 | 5 | Professional opportunities |
|  | Item: 211,222,223 & 224 | 4 | Praise & recognition |
|  | Item: 220,227,228, 229,230 &231 | 6 | Control & responsibility |
| Part-II | Job and organization related domain |  | - |
|  | Item: 250,229 &242 | 3 | workload |
|  | Item: 232,233 & 234 | 3 | Working condition |
|  | Item: 235,236,237,&238 | 4 | Supervision |
|  | Item: 239,240 & 241 | 3 | Achievements |
|  | Item: 243,244 &245 | 3 | Nature of work itself |
|  | Item: 247,248 & 249 | 3 | organization and administration policies |
|  | Item: 251,252,253,254& 255 | 5 | Standard of care |
|  | Item: 245, 250, 201 | 3 | Fairness of payment |
